# Exploration of COVID-19-Based Changes to Caregiver Burden and Caregiving Intensity among Informal Caregivers

**DOI:** 10.1093/geroni/igaa057.3459

**Published:** 2020-12-16

**Authors:** Steven Cohen, Zachary Kunicki, Megan Drohan, Mary Greaney

**Affiliations:** 1 University of Rhode Island, Kingston, Rhode Island, United States; 2 Brown University, Providence, Rhode Island, United States

## Abstract

Individuals providing unpaid care of assistance to family members and friends (e.g. informal caregivers), may have been uniquely impacted by the COVID-19 pandemic. Research is needed to examine the pandemic’s effect on informal caregivers’ caregiving intensity and burden. Therefore, this cross-sectional study was conducted to explore self-reported changes in caregiver intensity (CI) and caregiver burden (CB) due to the pandemic to identify factors associated with changes in responsibilities and burdens. In June 2020, informal caregivers providing care to someone aged 50+ (n=835) reported their current and pre-pandemic caregiving intensity and burden. Data were collected via Amazon’s Mechanical Turk. Chi-square tests were used to examine bivariate associations between pandemic time (pre vs. post) differences in CI and CB. Multinomial regression was used to assess multivariate predictors of changes to CI and CB due to COVID-19. Results showed a significant U-shaped association between initial CB and CB change due to COVID-19. Higher levels of initial CB were associated with both a significant decrease in CB during COVID-19 (OR 1.33, 95%CI 1.06-1.67), and a significant increase in CB during COVID-19 (OR 1.22, 95%CI 1.05-1.43). There were no significant associations between initial CB and changes in CI due to COVID-19, although older caregivers were more likely to experience a decrease in CB due to caregiving (OR 1.02, 95%CI 1.00-1.05). These mixed results suggest that caregivers with high initial CB experienced the most extreme changes to CB due to COVID-19. Future planned analyses will focus on understanding the potential drivers behind these unexpected results.

